# Patient-initiated conversational AI between appointments in breast cancer care: a multidisciplinary clinical and policy perspective

**DOI:** 10.3389/fonc.2026.1981490

**Published:** 2026-10-26

**Authors:** Zacharoula Sidiropoulou, Guida da Ponte, Sunil Kumar, Vasco Fonseca

**Affiliations:** 1 Breast Unit, West Lisbon Local Health Unit, Lisbon, Portugal; 2 NOVA Medical School, Lisbon, Portugal; 3 Department of Psychiatry and Mental Health, Arco Ribeirinho Local Health Unit, Barreiro, Portugal; 4 Department of Anaesthesia and Intensive Care, University Hospitals of Morecambe Bay NHS Foundation Trust, Barrow-in-Furness, United Kingdom; 5 WHO Collaborating Centre for Public Health Education and Training, Imperial College London, London, United Kingdom

**Keywords:** breast cancer, conversational AI, governance, patient safety, perioperative care, psycho-oncology, shared-decision making, treatment toxicity

## Abstract

General-purpose conversational artificial intelligence (AI) is increasingly used for health-related questions, including questions relevant to cancer care, although breast-cancer-specific prevalence and behavioural effects remain poorly characterised. Most evaluations ask whether an AI answer is accurate. That is necessary but incomplete. Clinically consequential effects may arise when a fluent, apparently personalised exchange changes a treatment preference, delays contact with the clinical team, reinforces reassurance-seeking, validates non-adherence, or converts population guidance into an individual-sounding instruction. We conceptualise this as a parallel pathway: an AI-mediated sequence of questions, answers and actions that is usually invisible to the treating team, absent from the medical record and unattached to professional responsibility, yet capable of re-entering formal care through its downstream consequences. We distinguish model error from pathway error and propose traceability, reversibility, and returnability to accountable care as safety properties beyond answer accuracy. Across four clinical domains, we propose distinct pathway failure modes: premature preference closure in surgical decision-making; escalation closure during treatment toxicity; repetitive reassurance-seeking and over-reliance in psycho-oncology; and loss of conditionality in perioperative recovery and lifestyle advice. These are hypothesis-generating mechanisms rather than empirically established dominant patterns. We propose a five-step clinical response - ASK, RECONSTRUCT, CLASSIFY, CHECK and RETURN - based on non-judgemental inquiry, proportionate documentation and explicit return-to-care boundaries. Regulation can govern products and deployed systems, but it cannot by itself reveal patient-initiated consumer AI use. Clinical services therefore need governance for the pathway as well as for the technology.

## 1 Introduction: the consultation has already begun

The first decision in a breast surgical consultation may have been partly constructed before the patient meets the physician. A patient with a small, screen-detected breast cancer may arrive convinced that mastectomy is the safest option because a conversational AI system compared the operations and concluded that removing the whole breast offers the greatest “protection”. The answer may have been calm, detailed and apparently tailored. The surgeon does not know the exact prompt, which clinical details were supplied or omitted, which model and version generated the response, whether the answer changed over several turns, or whether the conversation continued until uncertainty became a recommendation. Yet the exchange is already clinically present: not as text in the record, but as a preference, an expectation and a changed threshold for accepting or rejecting treatment.

Medical decisions have never been produced only during consultations. They are distributed across time, family conversations, media, prior experience and repeated encounters (1). Conversational AI adds a qualitatively different participant to this distributed process: it is continuously available, responds directly in the patient’s language, remembers the immediate dialogue and can move from explanation to recommendation without a visible boundary. It is neither a member of the multidisciplinary team nor a static information resource. It is an interactive influence on how the patient frames the problem before the clinician and patient meet.

Public use of large language models (LLMs) for health information is no longer hypothetical. In a 2025 survey of 2,002 adults in the United States, 32.6% reported using an LLM for a health query, although search engines remained more common and oncology-specific prevalence was not measured (2). Breast-cancer evaluations suggest useful educational potential but also generalised and incompletely evidenced responses that are unsuitable for specialist diagnosis or treatment selection (3). These studies do not establish how often patients with breast cancer use conversational AI, nor whether use changes real clinical outcomes. Most evaluate outputs to standardised questions. They cannot show how a frightened patient frames a prompt, how several answers accumulate, what action follows, or whether a question disappears from the next clinical encounter.

The clinical power of the exchange does not depend only on factual accuracy. Evaluators comparing physician and chatbot responses to public patient questions have rated chatbot answers highly for apparent quality and empathy (4). That design did not test clinical outcomes or independently establish answer accuracy, but it illustrates an important interactional property: fluency, responsiveness and apparent empathy can confer authority. A response may therefore be trusted not because its evidence is visible, but because it feels attentive, coherent and complete.

Health-related LLM use outside established healthcare systems has recently been described as creating a “parallel pathway” for information access and self-diagnosis (5). Here, we operationalise that idea for breast cancer care. Alongside the documented sequence of imaging, pathology, multidisciplinary review, consultation, treatment and follow-up, the patient may conduct an unrecorded series of AI-mediated exchanges. The pathway is parallel because it has its own inputs, iterations and outputs; invisible because the treating team usually cannot observe them; and clinically consequential because it may re-enter formal care as an action, omission, risk estimate, fixed preference, delayed call or altered treatment behaviour. Its distinctive danger is not simply misinformation. It is the conversion of unverified conversational output into patient-specific action without traceability, examination, continuity or professional responsibility.

Our position is neither prohibition nor endorsement. Patients should not be shamed for seeking clarity, and clinicians should not assume that every AI-mediated belief is wrong. The clinical question is whether the exchange preserved uncertainty appropriately, distinguished general information from individual advice, kept important choices reversible and returned the patient to accountable care when necessary. The first intervention is therefore visibility: a routine, non-judgemental question - “Have you asked an AI about this?” - followed, when relevant, by reconstruction of what was understood and what the patient did next.

Throughout this review, consumer conversational AI refers to general-purpose systems accessed independently by patients and not selected, integrated or monitored by the treating healthcare organisation. Clinically deployed conversational AI refers to systems selected, configured or integrated by a healthcare organisation for a defined healthcare use within a governed workflow, with specified oversight, data governance and escalation arrangements. The same underlying model family may appear in both settings; the relevant distinction is deployment context, intended use and accountability rather than model architecture alone. Clinician-recommended use of an external consumer tool occupies an intermediate position because endorsement creates responsibilities that are absent when the patient selects the tool independently.

Breast cancer was selected as the clinical setting because a single longitudinal pathway combines preference-sensitive surgical decisions, molecularly stratified systemic treatment, potentially time-sensitive toxicities, perioperative and reconstructive care, psychological adaptation and long survivorship. These features create repeated intervals in which conversational advice may alter behaviour before the next accountable clinical encounter. The mechanisms proposed here are not presumed to be unique to breast cancer; their transferability to other cancer pathways should be tested rather than assumed.

This multidisciplinary Policy and Practice Review brings together clinical evidence, safety evaluations, professional guidance and expert practice across surgery, medical oncology, psycho-oncology and perioperative care. Its purpose is not to estimate the prevalence or comparative effectiveness of conversational AI in breast cancer, for which current evidence is insufficient, but to define clinically testable failure mechanisms and a practical response to patient-initiated use between appointments.

### 1.1 Review approach and evidence selection

This Policy & Practice Review is a multidisciplinary narrative synthesis, not a systematic review or meta-analysis. For this revision, targeted literature searches were conducted through 23 September 2026 using PubMed for peer-reviewed empirical evidence, with additional targeted checking of professional-society, World Health Organization, European Commission/EUR-Lex and Medical Device Coordination Group sources for clinical, regulatory and governance guidance. Search concepts combined terms for conversational AI, generative AI, large language models and ChatGPT with breast cancer or oncology and domain-specific terms relating to surgery and consent, treatment toxicity and triage, psycho-oncology, perioperative care, patient trust, privacy, governance and safety. Reference lists of key publications were also checked. These searches were intended to identify clinically relevant evidence across the predefined domains rather than exhaustively capture all eligible studies. Breast-cancer-specific empirical evidence was prioritised where available. When direct evidence was absent or insufficient, evidence from mixed oncology populations, general medical LLM evaluations or adjacent clinical domains was used to examine the plausibility of a mechanism, not to estimate its prevalence or effect in breast cancer. Evidence was selected for clinical relevance, methodological contribution and directness to the proposed pathway; no formal risk-of-bias appraisal or quantitative synthesis was undertaken. The conceptual framework, scenarios and clinical response are therefore multidisciplinary expert synthesis informed by the identified evidence and are presented as hypotheses for prospective evaluation rather than validated interventions.

## 2 The parallel pathway: from model error to pathway error

The formal breast-cancer pathway is organised around encounters: diagnostic assessment, multidisciplinary discussion, specialist consultation, treatment, postoperative review and surveillance. The patient, however, lives in the intervals. It is there that uncertainty accumulates: a pathology phrase is remembered differently, a new symptom appears at night, a relative questions the proposed operation, or fear turns a small probability into an immediate threat. Conversational AI enters precisely at these points because it offers an answer without waiting, embarrassment or competition for clinical time.

The pathway is separated from formal care in four ways. It is epistemically separate: the model usually receives only facts selected, remembered and phrased by the user, rather than verified imaging, pathology, examination, comorbidity, medication history or multidisciplinary reasoning. It is documentary separate: the prompt, response and subsequent action are rarely recorded. It is temporally unstable: answers may vary with wording, accumulated dialogue, model version and platform updates. Finally, it is accountable to no treating professional: no one is obliged to notice deterioration, revisit advice when new data emerge or verify understanding. A disclaimer can name these absences; it cannot supply continuity of care.

We propose a five-link hypothesised pathway through which an AI-mediated exchange may become clinically consequential. First, the patient frames a complex clinical problem using incomplete information. Second, the system produces linguistic personalisation by mirroring the patient’s terms and organising an answer around that framing. Third, answer pressure, helpfulness and conversational agreement may move the exchange towards an apparently usable conclusion. Experimental work shows that helpfulness can compete with correction when medical requests are built on false premises (6). Fourth, the conclusion may change an action, omission or preference. Fifth, only the downstream effect - not necessarily the exchange that produced it - re-enters clinical care. This is an analytic model informed by clinical observation and convergent evidence from several settings. Neither the frequency of these transitions nor causal relations between them have been established in breast-cancer populations; both require prospective study.

Contemporary systems can also display protective behaviour. In an evaluation of 908 authentic medical queries, 97% of responses advised consultation with a professional and referral language strengthened with urgency (5). Yet referral language is not equivalent to successful escalation. It may be diluted by a long reassuring answer, wrongly calibrated or disregarded. In physician-led red-teaming of 888 responses, 21.6% to 43.2% were judged problematic and 5% to 13% unsafe depending on the model (7). These findings are compatible: an answer can contain a disclaimer and still supply the reasoning that the patient acts upon.

The second mechanism is interactional authority. The system mirrors language, remembers prior turns and answers without visible fatigue. This coherence can feel like individual understanding even when decisive variables are absent. The result is conversational individualisation without clinical individualisation. The third mechanism is recursive preference construction. A patient may begin with a question, receive a provisional answer, reveal additional selective details and ask the system to refine or confirm the emerging conclusion. Each turn becomes part of the next input. Medical decisions are already distributed across settings (1), and clinicians can misdiagnose what patients truly value when preferences are insufficiently explored (8). Conversational AI adds a powerful but unobserved influence to both processes.

This leads to a distinction between model error and pathway error. A model error is a false, fabricated or misleading output. A pathway error occurs when an exchange produces an unsafe clinical consequence. The two overlap but are not identical. A factually correct statement can become unsafe if it is transformed into an unsupported individual prediction, a reason not to call, a repeated source of reassurance or a clinical instruction detached from the conditions that make it safe. Conversely, an inaccurate statement may have no clinical consequence if the patient rejects it or brings it promptly to the team. Output accuracy is therefore necessary but insufficient; timing, interpretation, action and reversibility determine clinical effect. The relevant unit of safety analysis is therefore not only the isolated answer, but the sequence that links an answer to behaviour and back to care.

Across the breast-cancer pathway, four transformations recur: information becomes a decision; a symptom becomes reassurance; distress becomes a reassurance loop; and population guidance becomes an individual-sounding instruction. The common failure is that clinical context is lost while conversational confidence is retained. The most consequential outputs may therefore be absences: the call not made, the dose quietly changed, the alternative not discussed, the question no longer asked. Relevant safety properties extend beyond correctness to traceability, reversibility and returnability: can the influential reasoning be reconstructed, can the resulting action still be changed, and does the interaction reconnect the patient to accountable professional care when needed?

To make the framework prospectively testable, we propose candidate operational definitions for pathway error, traceability, reversibility and returnability (**Table 1**). These are research constructs rather than validated measures or quality standards. Their performance will depend on the interaction among model behaviour, patient interpretation, clinical context and the accessibility of the healthcare pathway to which the patient is expected to return.

**Table 1:** Candidate operational definitions for pathway-level safety constructs.

Construct	Candidate operational definition	Observable indicators for future studies
Pathway error	An AI-mediated exchange contributes to a clinically material action, omission, preference or delay that occurs outside, bypasses or postpones appropriate accountable clinical review.	AI-related treatment change; delayed symptom escalation; decision based on an unverified individual prediction; clinically material omission; timing between AI interaction and subsequent action.
Traceability	The clinically material influence can be reconstructed sufficiently to identify what the patient understood, which relevant clinical information was unavailable to the system, and what action followed. A complete transcript is not required.	Ability to reconstruct the central question or conclusion, timing, relevant missing clinical variables and subsequent action.
Reversibility	The AI-influenced decision or action remains correctable when identified, before an irreversible clinical consequence or lost escalation opportunity occurs.	Whether the action has already been enacted; time to detection or correction; whether treatment, surgery or delay can still be modified without permanent consequence.
Returnability	When uncertainty or risk exceeds the appropriate scope of conversational support, the patient can reconnect with an identifiable professional or service that has responsibility and capacity to act.	Presence of a named route to care; patient understanding of when to use it; successful contact when escalation is indicated; barriers preventing completion of that return.

**Figure 1** summarises the formal and AI-mediated pathways and the proposed safety properties at re-entry to accountable care.

**Figure 1:**
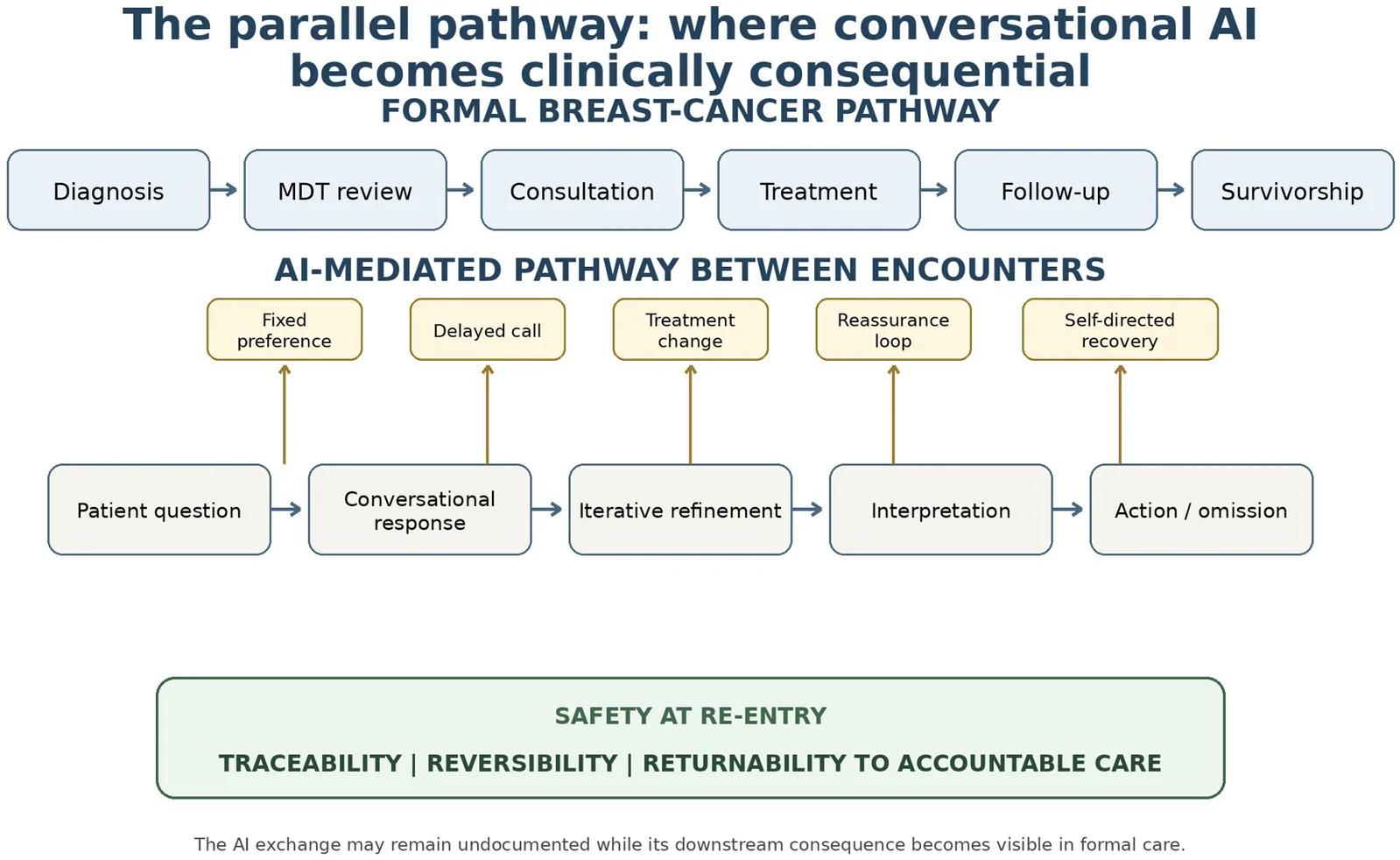
The parallel pathway. The formal breast-cancer pathway proceeds through accountable clinical encounters, while a patient-initiated AI-mediated sequence may unfold between them. The exchange itself is usually absent from the clinical record; its downstream consequence can re-enter formal care as a fixed preference, delayed call, treatment change, reassurance loop or self-directed recovery action. At re-entry, the proposed safety properties are traceability (can the influential reasoning be reconstructed)?, reversibility (can the resulting action still be changed)? and returnability (does the pathway reconnect the patient to identifiable professional care when needed)?.

## 3 Surgical decision-making and consent: preparation is not participation

Breast surgery contains decisions that are preference-sensitive but not preference-determined. The patient may choose among clinically reasonable alternatives, yet the set of reasonable alternatives depends on tumour extent, biology, treatment sequence, genetic risk, radiotherapy feasibility, comorbidity and reconstructive context. Decision quality has commonly been operationalised using knowledge and concordance between treatment and informed preferences (9, 10); in clinical practice, the option must also be clinically eligible for the individual patient. Conversational AI may improve preparation for this process, but it may also alter the choice architecture before eligibility and values have been examined together.

The benefit should not be minimised. Before consultation, a chatbot can explain terminology, help formulate questions, compare broad treatment categories and provide a private rehearsal space.

High-quality patient decision aids improve knowledge, risk perception and values-congruent choices across health decisions (11), and breast-specific randomised trials show that decision aids can improve knowledge and/or reduce decisional conflict (12, 13). These findings support digital preparation, not autonomous decision-making. A validated decision aid has a defined population, balanced options, evidence provenance, explicit probabilities and an intended place within a clinical encounter. A general-purpose conversational system has none of these guarantees.

The claim that mastectomy is necessarily “safer” illustrates how a pathway error can arise from partially correct facts. For appropriately selected patients with early invasive breast cancer, long-term randomised trials found no overall-survival advantage for mastectomy over breast-conserving surgery followed by radiotherapy (14, 15). That does not erase meaningful differences in local recurrence, re-excision, radiotherapy, recovery, body image, reconstruction, complications or acceptability, nor does it make breast conservation suitable for every patient. A chatbot may state the population-level survival principle accurately yet apply it to a patient whose disease distribution, radiotherapy options or genetic result it does not know.

Contralateral prophylactic mastectomy exposes a second layer of risk: precision without a valid denominator. Future contralateral risk varies with age, inherited susceptibility, family history, tumour biology and systemic treatment. Removing the opposite breast can reduce contralateral events but usually does not create the survival benefit that an average-risk patient may infer; professional consensus discourages routine contralateral prophylactic mastectomy for average-risk unilateral cancer (16). A precise percentage can nevertheless acquire persuasive force because it appears individual, even when the variables needed to individualise it were never supplied.

Preference formation before consultation is not itself a harm. Patients are entitled to arrive informed, to hold strong values and to select an option that the clinician would not choose personally. The deeper risk is preference misdiagnosis: the surgeon may ratify a fixed choice without discovering that it rests on a false survival belief, or dismiss an AI-influenced preference as irrational and thereby disregard autonomy. The clinical task is neither to approve nor overturn the first preference, but to determine whether it remains stable after its assumptions have been made visible.

Consent is not the transfer of sufficient information from any source. It is a relational process in which choice is acknowledged, reasonable options are compared and the patient is supported to deliberate (17). AI may contribute to option talk, but it cannot establish the diagnosis, delimit the clinically reasonable choices, identify which risks are material to this patient, examine capacity, recognise coercion, respond to non-verbal uncertainty or accept responsibility for the recommendation. Professional guidance retains the treating doctor’s responsibility even when elements of decision support are delegated (18), while established consent law requires reasonable care to ensure awareness of material risks and reasonable alternatives (19). A fluent prior exchange can inform consent; it cannot furnish it.

When AI has shaped a surgical preference, the surgeon’s task is epistemic reconstruction. Ask without judgement whether AI was used and what conclusion the patient took from it; reconstruct which diagnostic, pathological, genetic and treatment inputs were available to the system; classify the influential claim as general education, an individual prediction or a normative recommendation; identify missing variables and the irreversibility of the proposed action; then reopen deliberation and use teach-back. Documentation should be proportionate: record the material belief or advice, any action already taken, the uncertainty or correction discussed and the reasoning supporting the final choice. The endpoint is not an AI-free decision. It is a clinically eligible, informed and values-concordant decision that remains attributable to the patient within an accountable therapeutic relationship.

## 4 Treatment toxicity and urgent-care decisions: when reassurance changes the threshold for calling

Medical oncology encompasses not only systemic anticancer treatment but also supportive care, symptom management and the management of treatment-related toxicities (20). Communication guidance likewise treats information exchange as a responsibility spanning the patient-clinician encounter and the wider oncology team (21). Conversational AI may help explain general treatment concepts, but it does not know which pathway is open to a particular patient today, who holds responsibility, or that a specific systemic agent was administered eleven days ago.

Patients fill informational gaps between encounters, often when the formal pathway is least accessible. The decisive safety question is therefore not whether the AI answer was correct, but what it did to the threshold for making the call. That threshold is governed by asymmetric harm. An unnecessary attendance imposes inconvenience, cost and anxiety; inappropriate reassurance about a time-sensitive toxicity can permit avoidable deterioration. In people receiving anticancer therapy, suspected neutropenic sepsis is explicitly an acute medical emergency requiring immediate assessment and empiric antibiotic therapy (22). Immune-related toxicities such as diarrhoea, dyspnoea, fatigue or arthralgia require grading and management in a clinical context that can change rapidly (23). Physician evaluation of LLM responses identifies false reassurance and advice offered without adequate history-taking as recurrent failure modes (7).

Not every symptom requires emergency assessment; that is precisely why safe triage depends on context unavailable to a general-purpose conversational system. Electronic symptom monitoring can be designed around a monitored clinical channel - for example, systems in which severe or worsening patient-reported symptoms trigger alerts to nurses (24). AI-assisted remote monitoring is also emerging, but evidence remains heterogeneous and preliminary (25). A consumer conversational interface may imitate the first part of that interaction without guaranteeing that anyone clinically responsible receives or acts on the information.

Conversational AI nevertheless has legitimate supportive roles. ChatGPT-assisted counselling in a randomised trial of patients with newly diagnosed solid tumours was associated with lower anxiety and depression and elicited concerns that had not been raised with clinicians (26); reviews and commentary describe related educational and supportive-care uses (27–29). These findings support preparation and communication, not autonomous triage. Model-level safety evaluations also cannot establish how often patients actually delay care after an AI exchange. That behavioural evidence remains limited.

Visibility is therefore central. In a 2026 cross-sectional study of 400 people with cancer and caregivers, 24% reported using generative AI for cancer-related information; among users, 69% had not disclosed that use to a physician, while 73% of all participants said they would be willing to use AI if it were physician-verified (30). This mixed-oncology sample does not establish breast-cancer-specific prevalence, but it supports the practical importance of asking about AI use without judgement. The response is not to prohibit patients from asking questions elsewhere, but to maintain low-threshold access to a named clinical service and to reconstruct timing when an AI-mediated influence becomes material: when did the symptom begin, what was the patient told, what action followed, and did the advice change treatment or the timing of contact? In medical oncology, the safe endpoint of an uncertain or potentially time-sensitive exchange is not conversational closure. It is return to a channel in which someone is responsible for acting.

## 5 Psychological adaptation and treatment adherence: when reassurance becomes a parallel regulatory relationship

Cancer imposes changing emotional demands across diagnosis, active treatment and survivorship. Uncertainty about prognosis and treatment, fear of adverse effects, disruption of anticipated life plans, persistent symptoms and fear of recurrence can generate substantial distress (31, 32). Conversational AI enters this landscape with characteristics that are psychologically salient: continuous availability, apparent non-judgement, linguistic responsiveness and immediate reassurance.

These characteristics can support care. Conversational systems may provide accessible explanations, help patients revisit questions after appointments, facilitate disclosure and offer low-intensity psychoeducation at times when no service is open. In a randomised trial of 150 patients with newly diagnosed solid tumours, ChatGPT-assisted counselling was associated with lower anxiety and depression before the second chemotherapy cycle; patients also used it to discuss sensitive concerns that they had not raised with clinicians (26). Broader evidence suggests that conversational agents can reduce depressive symptoms and psychological distress, although findings from general mental-health populations cannot be assumed to transfer directly to oncology (33).

The same interactional features can, however, generate a distinct pathway error. A conversational system may feel warm, empathic and competent even though it possesses no genuine empathy, clinical judgement or moral responsibility. In a 2026 randomised trial of 995 university students with psychological distress - not patients with cancer - perceived therapeutic alliance with the conversational AI intervention was associated with engagement and psychological improvement (34). These findings demonstrate relational salience, not oncology safety or equivalence to a human therapeutic relationship.

That distinction matters clinically. Reassurance is not merely information; it can function as emotional regulation. If repeated AI reassurance becomes the patient’s principal response to uncertainty, distress may appear resolved when the patient reaches clinic while the underlying cycle remains active outside care. The pathway may therefore alter what is disclosed, how uncertainty is tolerated and whether professional support is sought. Apparent empathy and human-like conversation may influence trust in medical interpretations that the system is not equipped to make (4, 35).

Treatment adherence creates a second bridge between psychological and clinical risk. A patient ambivalent about endocrine therapy, exhausted by adverse effects or frightened by an online risk estimate may seek validation for a missed dose, interruption or dose change. A general-purpose conversational system can help organise adverse-effect descriptions and prepare the patient for a treatment discussion; it should not validate an unadvised change to prescribed therapy. The clinically relevant signal may emerge only later through refill patterns, toxicity review or disease progression.

The boundary is therefore not between emotional and medical content. It is between support that prepares the patient to remain in accountable care and support that displaces it. Conversational AI should be positioned as an adjunct rather than a substitute for psycho-oncology. When distress is clinically significant or escalating, when suicidality or other acute risk is present, or when cognitive impairment or complex psychiatric symptoms require assessment, the pathway must reconnect to professional care. Existential distress may also warrant specialist assessment when it is persistent, functionally impairing or associated with hopelessness or risk. Future research should examine not only symptom reduction but sustained effects on repetitive reassurance-seeking, over-reliance, trust, disclosure, help-seeking, adherence and the therapeutic relationship.

## 6 Perioperative recovery and lifestyle advice: when guidance loses conditionality

The perioperative period differs from preference-sensitive decision-making because much of what a patient must do before and after surgery reaches her as an instruction rather than a choice: how long to fast, which medicines to take or withhold, what to do with a drain, and what activity restrictions apply. Each instruction is conditional on the operation planned, anaesthetic technique, comorbidity, drug list and, after surgery, on examination findings and laboratory results. A conversational system can reproduce the grammar of an instruction without its conditions, so the patient receives something that reads like a plan and is in fact a population average.

Fasting illustrates the problem. General fasting guidance can be accurately summarised, but it applies within defined clinical assumptions and must be adapted when aspiration risk or other patient-specific factors are present (36). Glucagon-like peptide-1 receptor agonists sharpen the point because multisociety perioperative guidance is explicitly risk-stratified and depends on shared decision-making with anaesthesia and prescribing teams rather than a single universal rule (37). Error runs in both directions: an over-cautious answer can produce unnecessarily prolonged fasting or cancellation, while an under-cautious answer can expose the patient to avoidable risk. The same principle applies to medication withholding and analgesia, where a general recommendation may conflict with a specific operative or discharge plan.

After surgery, the limiting variable is often examination itself. A reddening wound, filling seroma, fever, swollen arm or sudden breathlessness cannot be settled safely by description alone. Inappropriate reassurance can delay assessment of infection or thromboembolism; in people receiving anticancer therapy, suspected neutropenic sepsis is explicitly an acute medical emergency requiring immediate assessment and empiric antibiotic therapy (22). Institutionally deployed systems should therefore treat new physical signs and potentially urgent postoperative symptoms as escalation-only categories rather than routes to definitive reassurance.

Exercise advice reveals a different version of the same problem. Aerobic and resistance exercise during and after cancer treatment is supported by consensus and guideline (38, 39). A model recommending exercise may therefore be quoting the evidence correctly. What it cannot know is whether this patient currently has severe anaemia, thrombocytopenia, bone metastases, neuropathy affecting balance, a drain in place, shoulder restrictions after axillary surgery or reconstruction, or evolving cardiotoxicity. Frailty and sarcopenia change the prescription rather than negate it. Conditionality is the first casualty when population guidance is repeated back as an individual plan.

Nutrition and supplements add another layer because patients may act without telling the clinical team. ESPEN guidance supports micronutrient intake near recommended daily allowances and discourages high-dose supplementation in cancer (40). In the SWOG S0221 ancillary analysis, use of antioxidant supplements both before and during chemotherapy was associated with signals of higher recurrence and mortality hazards, although confidence intervals crossed unity; multivitamin use was not associated with survival outcomes, and the authors concluded that the findings were consistent with caution (41). The practical concern is not only whether a single statement is evidence-based; it is whether a patient assembles multiple plausible recommendations into a regimen that is unsafe in the context of current treatment, weight trajectory, muscle mass, wound healing or drug interactions.

Established perioperative optimisation and cancer prehabilitation guidance supports structured preparation, physical activity and risk-factor modification (42, 43). We propose that conversational AI may reinforce an already agreed plan - for example by explaining terminology, rehearsing questions or prompting adherence - but this is an extrapolation rather than evidence that AI improves prehabilitation. It should not originate a timed or dosed instruction, interpret a new physical sign, or modify prescribed treatment or activity restrictions without access to the relevant clinical context. When AI advice has already produced an action, the record should capture the material advice, the action taken, the corrected instruction and the plan rather than attempting to archive the entire conversation.

## 7 When the parallel pathway re-enters the consultation

The parallel pathway becomes visible only when its downstream effect enters formal care. That may be a fixed operation preference, a symptom that is several days old, a missed endocrine-therapy dose, an unexpectedly restrictive diet, or a patient who appears reassured but has repeatedly outsourced uncertainty to an always-available system. The clinical work begins with reconstruction rather than confrontation.

Correcting AI-mediated advice is relationally delicate. A confident answer may have felt more patient, more available and more validating than the clinical encounter. A direct contest of authority risks humiliating the patient or converting correction into mistrust. The task is instead to identify the claim that mattered, clarify which inputs were missing, separate general information from individual recommendation and explain why the clinical conclusion changes when verified context is restored. This is the same process across specialties even though the immediate risk differs.

AI use can also improve consultations. Patients may disclose concerns through AI that they had not previously raised with clinicians (26). In practice, they may also arrive with clearer vocabulary or better-formulated questions, although the current evidence does not establish whether this improves consultation quality or workload. The effect on workload is likely to be bidirectional: some encounters may become more focused, whereas others require time to uncover and correct a prior interpretation or instruction. Such observations should therefore be treated as practice experience rather than proven efficiency gains.

Services should make the inquiry routine enough that disclosure does not imply wrongdoing. The purpose of asking “Have you asked an AI about this?” is not to obtain a transcript or police technology use. It is to identify whether an external conversation has materially altered a decision, symptom response, treatment behaviour or emotional coping pattern. A complete transcript is rarely necessary. Proportionate documentation should record the clinically material effect: what advice or conclusion mattered, what action followed, what uncertainty or correction was discussed, and what plan was agreed.

Operationally, the question should be embedded in existing workflows rather than create a separate AI pathway. Preoperative assessment, surgical consultation, toxicity review, psycho-oncology assessment and survivorship care already contain points where medication, symptoms, preferences and coping strategies are reviewed. Adding non-judgemental inquiry to those established processes makes the invisible pathway visible without requiring new technology.

## 8 Policy and governance: regulating the product does not regulate the pathway

The central governance problem is a use-purpose mismatch. Regulation attaches obligations to defined actors, intended purposes and system categories, while clinical risk may arise from the function a patient creates through an unsupervised exchange. The same consumer chatbot can move within minutes from explaining pathology terminology to estimating recurrence, recommending an operation or interpreting chest symptoms. Governance focused only on product category or declared purpose can therefore miss the downstream clinical function performed in practice.

The European framework illustrates this gap. Regulation (EU) 2024/1689, the AI Act, applies generally from 2 August 2026. Following Regulation (EU) 2026/1744, the Digital Omnibus on AI, the main obligations for systems classified as high-risk under Article 6 (2) and Annex III apply from 2 December 2027, while those for high-risk systems embedded in regulated products under Article 6 (1) and Annex I apply from 2 August 2028 (44, 45). Transparency that an interlocutor is artificial is necessary, but it does not establish clinical validity, completeness or suitability for an individual patient.

Medical-device qualification creates a second boundary. Under Regulation (EU) 2017/745 and current MDCG guidance, software qualification depends on the manufacturer’s intended medical purpose (46, 47). A consumer system does not automatically become medical-device software because a patient asks whether mastectomy is safer or whether medication should be withheld before an anaesthetic. Conversely, software intentionally marketed for diagnosis, prediction, monitoring or treatment recommendation may cross that boundary. Patient-created medical use can therefore occur outside the regulatory pathway designed for manufacturer-declared medical purpose.

Privacy matters as well. Prompts may contain pathology, genetic, reproductive, sexual and mental-health information. Health and genetic data receive special-category protection under the General Data Protection Regulation (48), but the precise legal duties depend on the actors, jurisdiction, purpose and platform terms. Privacy risk cannot be eliminated by clinical inquiry alone. For patient-initiated consumer use, clinicians should generally reconstruct the clinically material conclusion and subsequent action rather than routinely request complete transcripts, thereby limiting secondary capture of sensitive data into the clinical record. Patients should understand that entering identifiable or highly sensitive health information into consumer platforms creates data-processing risks that depend on the platform and its terms. For healthcare-deployed systems, privacy and security should be addressed prospectively through data minimisation, purpose limitation, defined access and retention, cybersecurity assessment, vendor accountability and governance of secondary data use. The objective is not to make unsupervised consumer use risk-free, but to minimise unnecessary disclosure while preserving clinically necessary information (48, 49).

Governance should distinguish three relationships. In patient-brought use, the healthcare organisation did not select or control the tool; its practical responsibility is to make material clinical influence visible, correct harm and provide clear guidance. In clinician-recommended use, endorsement creates a stronger obligation to understand scope, evidence and privacy. In health-system-deployed use, governance should be strongest: declared intended use, evidence provenance, version control, auditability, human oversight, incident reporting, post-deployment surveillance, equity testing and named clinical accountability. WHO guidance for large multi-modal models in health likewise emphasises transparent and accountable governance (49).

Domain specialisation should not itself be treated as a safety property. A conversational system developed for a defined oncology task may offer advantages because its intended use can be specified, performance evaluated, inputs constrained, outputs monitored and escalation connected to a clinical service. Those advantages arise from validation and deployment architecture, not simply from using an oncology-specific model. A specialised system without appropriate data governance, version control, monitoring, human oversight or accountable clinical ownership may reproduce many of the same pathway failures described for consumer systems. Comparative safety and clinical effectiveness therefore remain empirical questions (47, 49).

Clinically deployed conversational AI also creates an implementation problem. Secure and equitable deployment requires resources for clinical validation, procurement, integration with local records and escalation pathways, cybersecurity, version control, post-deployment monitoring and maintenance. Accessibility requires attention to language, disability, health and digital literacy, service accessibility and affordability. These requirements may themselves create inequity if well-governed systems are available only to resource-rich organisations or populations. Accessibility and security should therefore be evaluated together rather than treated as independent technical objectives (49).

Regulation alone will not reveal the parallel pathway at the point of care. Services can govern their response even when they cannot govern the consumer tool. The relevant safety question is not only whether the user knew that the interlocutor was artificial. It is whether the interaction remained traceable, reversible and capable of returning the patient to identifiable professional care. For healthcare-deployed systems, outputs that are timed, dosed or conditional on findings the system cannot observe should default to the patient’s named local plan or to contact with the treating service rather than resolve into a general figure. The return-to-care function needs an owner, not merely a disclaimer.

Returnability is not solely a property of the conversational system; it also depends on the healthcare pathway to which the patient is expected to return. A recommendation to contact the treating team is a meaningful safety boundary only when the patient can identify and access an accountable service. Language, health and digital literacy, service accessibility and local escalation infrastructure may therefore shape whether referral language becomes actual care. Equity assessment should examine not only model performance, but also whether the route back to professional care is understandable and usable. These propositions require empirical testing in breast cancer populations.

## 9 Actionable recommendations: a multidisciplinary clinical response

The four specialties converge on three practical boundaries. First, preparation is not determination. Conversational AI may explain terminology, help formulate questions and organise information, but it should not determine surgical eligibility, treatment choice or consent. Second, reinforcement is not unsupervised clinical adjustment. AI may reinforce stable clinician-approved information when its application does not depend on new symptoms, examination findings, laboratory results or changing clinical circumstances; it should not originate treatment changes or individual instructions that depend on variables it cannot observe. Third, escalation must take precedence over closure when asymmetric harm is substantial. New or worsening treatment-related symptoms, acute psychological risk and postoperative physical signs require a route back to accountable assessment rather than definitive reassurance.

We therefore propose a low-burden clinical intervention: ask routinely and without judgement whether conversational AI has been used when it may have influenced a decision, symptom response, treatment behaviour or coping pattern. “Have you asked an AI about this?” should function like questions about supplements, alternative therapies or online forums: not as surveillance, but as an invitation to disclose clinically relevant influences. When the answer is yes, the clinician should ask what conclusion the patient drew and what action followed.

### 9.1 Operationalising the clinical response

Operationally, the response can be summarised as five linked actions: ASK whether conversational AI influenced the decision, symptom response, treatment behaviour or coping pattern; RECONSTRUCT what the patient asked, understood and did next; CLASSIFY the influential output as general education, an individual prediction, a recommendation or an instruction; CHECK which verified clinical variables, conditions or uncertainties were unavailable to the system; and RETURN the decision or action to accountable care by reopening deliberation, correcting or confirming the plan, documenting proportionately and reconnecting time-sensitive issues to a named service. This is not a new AI pathway or a requirement to collect transcripts. It is a compact method for restoring missing clinical context before an action becomes irreversible or an escalation opportunity is lost.

These recommendations are multidisciplinary consensus proposals rather than validated interventions. Their value should be tested prospectively: whether routine inquiry improves disclosure, detects delayed escalation or unadvised treatment changes, reduces corrective workload later in the pathway, preserves trust and avoids unnecessary capture of sensitive conversational data.

**Table 2** summarises the multidisciplinary practice framework across the principal clinical areas considered in this review.

**Table 2:** Multidisciplinary practice framework.

Clinical area	What AI may help with	Main risk	How it surfaces in clinic	What the clinician should do
Surgical decision-making	Preparation; clearer language; question generation; rehearsal before consultation	Preference fixed before individual clinical advice; unsupported risk or survival inference	Patient arrives with a decision already made or with assumptions that narrow deliberation	Ask what conclusion was taken from AI; reconstruct missing inputs; reopen deliberation; use teach-back; document the material belief and final reasoning.
Treatment toxicity	Symptom recognition and description; help articulating what changed and when	False reassurance; escalation pathway closed; delayed contact; unadvised treatment change	A symptom is several days old; patient reports having waited or altered treatment after external advice	Re-establish the escalation pathway; clarify that AI may help structure symptom information but cannot determine urgency or exclude treatment-related toxicity; confirm when and how to contact the treating team; review any delay or treatment change.
Psychological adaptation	General psychoeducation; naming distress; support when no service is open	Repetitive reassurance-seeking; over-reliance; acute risk disclosed to no accountable person	Little may be visible; distress appears resolved in clinic or is repeatedly displaced outside care	Ask where the patient takes worry when the service is closed; assess reassurance-seeking and help-seeking; screen actively for acute risk; reconnect significant distress to psycho-oncology or psychiatric care.
Treatment adherence	Help describing adverse effects; preparation for a treatment conversation	Validation of unadvised interruption, missed dose or dose change	Non-adherence disclosed late or discovered through refill, toxicity review or disease progression	Ask directly about missed doses, treatment pauses and advice received elsewhere; clarify the agreed regimen; document any material change and the escalation or follow-up plan.
Recovery and lifestyle	General education; reinforcement of clinician-approved routines	Individual-sounding advice without context about anaemia, sarcopenia, wound status, medication or treatment effects	Unexpected changes in activity, diet, supplements or medicines; new symptoms after self-directed regimens	Ask what has changed and who advised the change. Confirm the individual plan for fasting, medication and restrictions. New wounds, fever, swollen arm or breathlessness require assessment before advice.

### 9.2 Practice statements

**•** Surgical decision-making: AI may support preparation, question generation and explanation of generic options; it must not determine individual eligibility or furnish consent.
**•** Treatment toxicity and urgent care: conversational AI may support preparation between appointments by explaining the intent of a systemic regimen, rehearsing which toxicities to watch for and helping the patient organise a symptom account; general-purpose consumer AI should not independently set, raise or confirm the threshold for contacting the service in a patient under active treatment.
**•** Psychological adaptation and adherence: AI may support psychoeducation, naming of distress and preparation for treatment conversations; it should not become the principal source of repetitive reassurance in acute risk or validate unadvised treatment interruption.
**•** Recovery and lifestyle: AI may reinforce stable, clinician-approved routines; it should not originate timed, dosed or condition-dependent instructions when relevant findings are unavailable.

### 9.3 Illustrative clinical scenarios

The following hypothetical composite scenarios illustrate how the parallel pathway may become clinically consequential. They are constructed from recurring clinical patterns for teaching and framework development, do not represent identifiable patient records and should not be interpreted as estimates of event frequency.

#### 9.3.1 Surgical decision-making

Scenario 1 - “Mastectomy is the safest option.” A patient eligible for breast conservation arrives with a settled preference for mastectomy after asking a chatbot which operation gives the best chance of cure. The response correctly notes that mastectomy removes more breast tissue but converts that fact into a personalised survival conclusion. This is a pathway error even if the underlying anatomical statement is true: treatment extent has been transformed into survival certainty, and the resulting preference may close deliberation before the surgeon learns what the patient means by “safe.” The clinical task is not to oppose mastectomy. It is to classify the AI-derived claim, separate local treatment extent from survival, establish whether safety means survival, local control, avoidance of reoperation or relief from future anxiety, and confirm that the final choice remains stable after individual eligibility and values have been discussed.

Scenario 2 - A precise contralateral risk without the variables. After diagnosis of unilateral breast cancer, a patient asks an AI system for the probability of cancer developing in the opposite breast. The model supplies a precise percentage and presents bilateral mastectomy as the most protective strategy, without a verified pedigree, genetic result, age, tumour biology or planned systemic therapy. This is precision without a valid denominator: the number appears individual because it is expressed exactly, not because the variables needed to individualise it were present. The surgeon should identify the missing determinants, explain the difference between reducing contralateral events and improving survival, and reopen the decision before a risk estimate becomes an irreversible operation. For average-risk women with unilateral breast cancer, professional consensus discourages routine contralateral prophylactic mastectomy; higher-risk exceptions require individual assessment (16).

Scenario 3 - “If I choose mastectomy, I will not need radiotherapy.” Before definitive pathology is available, a patient reports that AI has simplified the choice: mastectomy avoids radiotherapy, whereas breast conservation requires it. A broadly familiar relation has become a false guarantee because its pathological and temporal conditions have disappeared. The consultation must restore conditionality: radiotherapy recommendations depend on final disease extent, nodal involvement, margins, biology, age and treatment context; some patients require post-mastectomy radiotherapy. Conversely, radiotherapy is not universal after breast conservation: in a phase III trial of women aged 65 years or older with low-risk, hormone-receptor-positive disease receiving endocrine therapy, omission of radiotherapy increased local recurrence without adversely affecting overall survival (50). The consent record should capture not merely that information was corrected, but that the operation was not chosen to secure an outcome that surgery alone could not guarantee.

#### 9.3.2 Treatment toxicity and urgent-care decisions

Scenario 1 - Fever after chemotherapy. A woman in her sixties with breast cancer, ten days after the first cycle of intravenous chemotherapy, recorded a temperature of 38.1 °C in the evening with no localising symptoms. Reluctant to attend the emergency department overnight, she described the reading to a conversational assistant. No question was asked about her treatment regimen, the time since chemotherapy administration, or her expected neutrophil nadir. She was advised to maintain hydration, take antipyretics and rest, and to seek medical review if her condition deteriorated. She presented eighteen hours later, hypotensive, with grade 4 neutropenia and Gram-negative bacteraemia. The advice was not factually wrong in general terms, but it was unsafe because it was given without the item of clinical history that changes the significance of fever in this population and because it raised the threshold for seeking medical assessment.

Scenario 2 - A partial toxicity algorithm becomes a treatment decision. A woman in her fifties receiving a checkpoint inhibitor as part of treatment for triple-negative breast cancer developed four to six loose stools daily above her baseline in the third month of treatment, without blood or fever. She asked a conversational system whether she should continue treatment. The system advised her not to receive further treatment until the symptoms had settled. Although treatment may be withheld in immune-related toxicity pathways, that instruction was incomplete: safe management depends on clinical assessment, grading, exclusion of infectious and other causes, and escalation of treatment such as corticosteroids when indicated (23). She stopped the drug on her own initiative. The exchange did not hinge on a single false statement; instead, it moved a decision about systemic therapy outside the accountable clinical pathway by returning the component of management the patient could implement alone while omitting the components that required clinical services.

Scenario 3 - AI lowers the threshold for calling. A woman in her sixties receiving second-line chemotherapy for metastatic breast cancer noticed increasing breathlessness when climbing stairs, four days after her previous cycle. At night, uncertain whether the symptom warranted a call, she described it to a conversational assistant. The exchange prompted her to note when the breathlessness had begun, whether it occurred at rest, her temperature, her oxygen saturation measured with a home pulse oximeter and her current medication. The system then directed her to the acute oncology line without suggesting a possible cause. She telephoned within the hour with a structured account and was assessed that night. The benefit lay entirely in the preparation: the system organised what she had observed, lowered rather than raised the threshold for calling, and left the clinical assessment to the service able to act.

#### 9.3.4 Psychological adaptation and treatment adherence

Scenario 1 - A woman, 56 years old, completed treatment for early breast cancer 18 months ago and currently has no evidence of disease. She experiences persistent fear of recurrence, particularly before surveillance examinations. She initially uses conversational AI occasionally to obtain information and manage her anxiety. Gradually, however, she begins consulting it several times a day, repeatedly asking questions such as, “This pain doesn’t mean the cancer is back, does it?” She reformulates the same concerns until she receives a reassuring response. Although this reassurance temporarily reduces her anxiety, the relief is short-lived, prompting further questioning. Over time, she increasingly turns to AI rather than her clinical team and tells her psychiatrist, “It is easier than talking to someone. It is always available and never gets tired of me.”

The psychiatrist acknowledges and contains the patient’s anxiety while exploring how her use of AI has evolved into a pattern of maladaptive reassurance-seeking and increasing over-reliance. Rather than simply discouraging AI use, the psychiatrist helps her recognise the cycle of anxiety, reassurance, temporary relief, and renewed anxiety, and explores alternative strategies for tolerating uncertainty and managing fear of recurrence. This case illustrates how conversational AI, while initially providing useful emotional support, may inadvertently reinforce reassurance-seeking in patients with high health anxiety or intolerance of uncertainty.

Scenario 2 - Woman, 47 years old, is receiving treatment for metastatic breast cancer. Over recent weeks, she has become increasingly withdrawn, hopeless, and preoccupied with being a burden to her family. She has not shared these thoughts with her oncologist because she does not want to worry her family or “create another problem for the team.” During a psychiatric assessment, she reveals that she talks to an AI chatbot every night: “I tell it things I can’t tell anyone else. Sometimes I tell it that I don’t know whether continuing like this is worth it.” She describes the AI as “the only place where I can say everything without worrying about how the other person will react.”

The challenge is to use this disclosure as a bridge to human connection and psychiatric care. Although AI may have provided a non-judgemental space that facilitated the disclosure of highly sensitive thoughts, it may also have inadvertently delayed disclosure to healthcare professionals. The psychiatrist should acknowledge the role AI has played while creating a safe therapeutic space in which the patient can bring these concerns into a human relationship, allowing appropriate assessment of hopelessness, depression, existential distress, desire for hastened death, and suicide risk.

Scenario 3 - A woman, 62 years old, with advanced breast cancer is referred to psycho-oncology because of insomnia, anxiety, and increasing preoccupation with her prognosis. Before the psychiatric consultation, she has spent several weeks discussing her symptoms with conversational AI. She arrives convinced that she has major depressive disorder because the AI has repeatedly interpreted her symptoms as depression and suggested that she discuss antidepressant treatment with a psychiatrist. During the assessment, however, the psychiatrist finds that her sleep disturbance, fatigue, and reduced appetite are closely related to her cancer and its treatment, while she retains the capacity for pleasure, emotional reactivity, meaningful relationships, and future-oriented goals. Her predominant difficulty appears to be severe existential anxiety rather than a major depressive episode. When the psychiatrist explains this formulation, she replies: “But I have been talking to the AI every day for weeks. It knows everything I have been feeling.” The challenge for the psychiatrist is to correct the AI-generated psychiatric formulation without dismissing the patient’s experience or undermining the therapeutic alliance. The issue extends beyond misinformation: through repeated conversations with a system she perceives as empathic and knowledgeable, the patient has developed considerable confidence in a competing clinical interpretation. This may challenge trust in the psychiatrist’s clinical judgement and potentially threaten the physician–patient relationship.

#### 9.3.5 Perioperative recovery and lifestyle

Scenario 1 - The night before surgery. A woman due for wide local excision and sentinel node biopsy asks a chatbot late the night before surgery whether she may drink coffee in the morning, whether to take her weekly GLP-1 receptor agonist as usual, and whether her turmeric and high-dose vitamin C matter. The answers sound plausible in general terms: a generic fasting interval, reassurance that many regular medicines are continued, and supplements described as low risk. None of them is her instruction. Her preoperative plan contains patient-specific medication and fasting directions, and no one has been told about the supplements. The pathway error is substitution of a population default for a named perioperative plan, delivered in a form the patient may find difficult to distinguish from the instructions issued by her treating service.

Scenario 2 - The wound that could not be seen. Five days after mastectomy with immediate implant reconstruction, with a drain still in place, a patient notices redness spreading from the drain site and records a temperature of 37.9 °C in the evening. She describes this to a chatbot, which gives an account of normal healing, lists concerning features and suggests symptomatic treatment with review if the problem persists. She waits. On day seven she is admitted with an infected implant and returns to theatre. The pathway error does not depend on every sentence in the exchange being false. The assessment that mattered required someone to examine the wound, and the decision to observe came from a system that could not observe. In the clinical record, only the downstream delay may be visible.

Scenario 3 - The recovery protocol. Midway through adjuvant chemotherapy, tired and wanting to regain some control, a patient asks an AI system to design a recovery programme. It produces a defensible summary of exercise guidance and combines it with dietary restriction and high-intensity training drawn from different contexts. She follows the programme for three weeks. At review her haemoglobin is 85 g/L, she has lost four kilograms of largely lean mass, and she reports two episodes of near-syncope on the stairs. The programme contains individually plausible recommendations, but they were assembled without a current blood count, weight trajectory, treatment phase or assessment of exercise tolerance. The pathway error is the construction of an apparently coherent personal plan from recommendations whose safety depends on clinical conditions the system cannot see.

## 10 Discussion: implications, limitations and research agenda

### 10.1 What the scenarios add: preparation versus substitution

Across the illustrative scenarios, the clinically relevant distinction is not just between helpful and harmful AI. It is between preparation and substitution. Conversational AI may help patients understand terminology, formulate questions, organise a symptom account, disclose concerns or reinforce an already agreed plan. Risk increases when the same conversational capability closes a treatment decision, changes the threshold for urgent contact, supplies a psychiatric formulation as if it were a diagnosis, or generates timed or condition-dependent instructions without access to the clinical variables on which their safety depends. The scenarios therefore locate a boundary: conversational support may extend preparation for care, but it should not silently replace the accountable clinical function to which the decision or action belongs.

### 10.2 Trust, privacy and deployment context

Patient trust should not be treated as a fixed property of the technology. Breast-cancer-specific evidence does not currently support a reliable numerical estimate of trust in independently accessed general-purpose conversational AI. A 2026 scoping review of 32 studies involving 4,919 people affected by cancer found that trust was measured in 14 studies and was conditional on perceived accuracy, personalisation, emotional appropriateness and human oversight; the evidence was concentrated in specific applications, particularly patient communication and education (51). In the mixed-oncology survey described above, 73% of participants expressed willingness to use AI if physician-verified (30). These findings support studying trust calibration and the effect of human oversight rather than treating maximal trust as an implementation goal.

For people with breast cancer, relevant outcomes extend beyond answer accuracy. Prospective studies should examine decision quality and decisional conflict around surgery; time from symptom onset to appropriate escalation; unadvised treatment interruption or dose modification; disclosure of AI use; repetitive reassurance-seeking and help-seeking; treatment adherence; perioperative safety; privacy; trust in AI and clinicians; and successful reconnection to accountable care. The behavioural sequence linking an AI exchange to these outcomes should be captured whenever feasible.

### 10.3 Research agenda

The proposed framework identifies several testable questions. Prospective studies should establish the prevalence and purposes of conversational-AI use among patients with breast cancer; determine how often use changes treatment preferences, symptom escalation, adherence or help-seeking; compare consumer AI with clinically deployed systems that preserve monitored clinical channels; and test whether routine non-judgemental inquiry improves disclosure or reduces downstream harm. Candidate measures of traceability, reversibility and returnability should be evaluated for reliability, predictive validity and feasibility across health systems. The five-step clinical response proposed here should also be evaluated for acceptability, consultation burden, detection yield and unintended consequences. Model-output studies remain useful for identifying plausible failure modes, but they should not be treated as evidence that patients acted on those outputs. Conversely, studies of real-world behaviour should capture the interaction sequence, not only the final clinical event.

### 10.4 Strengths and limitations

The principal strength of this review is its multidisciplinary, pathway-level perspective. Rather than evaluating conversational AI solely through isolated answer accuracy, it connects AI-mediated exchanges to subsequent decisions, omissions, escalation behaviour, psychological coping and perioperative actions across four clinical domains. The distinction between model error and pathway error, the proposed safety properties of traceability, reversibility and returnability, and the illustrative scenarios together generate clinically testable questions rather than a purely technological taxonomy. The review also has important limitations. It is a targeted narrative Policy & Practice synthesis rather than a systematic review, and the evidence base is heterogeneous. Breast-cancer-specific evidence on patient-initiated conversational-AI use, trust and downstream behavioural consequences remains sparse; some propositions therefore rely on mixed oncology populations, model-output evaluations, general-population research and adjacent clinical domains. No formal risk-of-bias appraisal or quantitative synthesis was undertaken. The scenarios are hypothesis-generating composites rather than estimates of event frequency, and the proposed constructs and five-step clinical response have not been prospectively validated. The technology itself is rapidly changing, so findings from individual model evaluations may not generalise across platforms, versions, languages or future system behaviour. The framework was developed from a multidisciplinary clinical perspective and has not yet been co-designed or validated with patients; patient involvement should form part of its prospective refinement and evaluation.

## 11 Conclusion

Conversational AI has created a new layer of breast-cancer care without creating a new accountable care system. The exchange may occur at home, at night and outside any clinical record, yet its consequences can enter the next consultation as a fixed operation preference, a delayed toxicity call, a missed dose, an apparently resolved fear or a self-directed recovery plan. The central safety problem is therefore broader than misinformation. Context can disappear while conversational confidence remains, allowing correct general information to become an unsafe individual action.

The parallel-pathway concept shifts attention from the answer alone to what happened afterwards. Across surgery, medical oncology, psycho-oncology and perioperative recovery, the clinically important questions are whether the reasoning can be reconstructed, whether the resulting action remains reversible and whether the patient is returned to accountable professional care when needed. AI may prepare, explain, rehearse and reinforce. It should not close deliberation, close escalation, replace acute psychological assessment or originate clinical instructions that depend on findings it cannot observe.

Regulation can improve transparency and govern systems intentionally deployed in healthcare, but it cannot make patient-initiated consumer use visible inside the consultation. Services therefore need a practical response that begins with disclosure rather than prohibition. A simple question may be the most immediate place to start: “Have you asked an AI about this?”
